# Teaching Styles in Physical Education: A New Approach to Predicting Resilience and Bullying

**DOI:** 10.3390/ijerph17010076

**Published:** 2019-12-20

**Authors:** Carlos Montero-Carretero, Eduardo Cervelló

**Affiliations:** Department of Sport Sciences, Sport Research Center, Miguel Hernández University, 03202 Elche, Spain; ecervello@goumh.es

**Keywords:** teaching style, physical education, adolescence, bullying, resilience

## Abstract

The main objective of this study was to analyze student-perceived teaching styles’ power to predict students’ resilience and the emergence of bullying behaviors in physical education class. A total of 537 students of both sexes, between 11 and 15 years of age, from primary and secondary schools in the province of Alicante (Spain), participated in the study. The design of the study was cross-sectional. The results showed that bullying was positively predicted by students’ perceptions of a more controlling style and negatively by a greater perception of an autonomy-supportive style in physical education classes. Victimization was negatively predicted by greater resilience and positively by students’ perception of a teacher’s more controlling style. Finally, the mediation analysis showed that the perception of autonomy support indirectly and negatively predicted victimization, with resilience acting as a mediator. These findings provide useful information for physical education teachers interested in preventing bullying, and have important practical implications about the teaching style recommended for this purpose.

## 1. Introduction

Teachers’ intervention is determinant for the integral formation of students. According to Gilar-Corbi, et al. ([1], p. 161) “*the goal of a complete education is not only to teach technical skills, but also other skills, such as teamwork, effective communication skills, project and time management, and the ability to coordinate our emotions for that purpose*.” Thus, it is difficult to consider the success of adolescents’ education process without considering it from a broad perspective and addressing the main problems faced by students. In this sense, bullying has become one of the main pitfalls to be overcome by the educational community, concerned with its prevention [2,3] due to its high presence in schools worldwide [4,5], and its devastating consequences in the affected youths’ health [6]. A meta-analysis [7] showing 80 international studies indicated prevalence levels of 34.5 and 36% for bullying perpetration and bullying victimization, respectively. Available data on bullying in Spain for adolescents aged 12 to 18 in Compulsory Secondary Education show that between 3 and 5% of the students are victims of severe bullying, whereas between 15 and 20% suffer moderate bullying [8]. Although “*for many years, schools in Spain have maintained a passive stance toward teen bullying, with the understanding that the role of teachers was to teach, rather than participate in mediation in these cases*” ([8], p. 594), considering the prevalence data and some cases of great social impact that have ended in suicide of students who suffered bullying in Spain, there is a currently growing concern to determine what teachers and other school staff can do to combat bullying.

About forty years of research on this topic have advanced our knowledge of the characteristics that define this particular type of violence, as well as of some of the predictor variables of these behaviors. However, further analysis of the relationships between the variables involved seems necessary to develop more effective strategies that increase the effectiveness of intervention programs [9]. The inclusion of new or understudied variables in the predictive models, as well as methodological approaches that reveal hidden relationships between the antecedents of bullying, could help achieve this objective. This paper aims to fill some gaps in the literature of the study of the hidden relationships between variables, including the study of resilience as an antecedent, as some researchers demand [10]. In addition, considering the responsibility given to the field of physical education (PE) in Spain for education in values and development of social and citizens’ competence [11], this work seeks to contribute to understanding the role of PE teachers regarding bullying.

Bullying is any unwanted aggressive behavior(s) by another youth or group of youths who are not siblings or current dating partners that involves an observed or perceived power imbalance and is repeated multiple times or is highly likely to be repeated. It implies the intention of causing physical, psychological, social or educational harm [12]. Thus, victims can be beaten, insulted, threatened, socially excluded, or suffer damage to their property, they feel helpless in the presence of other peers, known as spectators, who adopt multiple roles depending on their attitude toward the event of intimidation [13] and who become very important in the process [14].

Personal factors such as empathy, self-esteem, moral disengagement, depression, and anxiety, among others, have been indicated as antecedents of bullying and victimization behaviors [15]. By contrast, few works in the educational field have included the concept of resilience as a personal factor related to bullying [16].

Resilience is defined as the ability to recover and maintain adaptive behavior after an initial incapacity in the face of a stressful event. Rather than invulnerability to stress, it is the ability to recover from the effects of negative events [17]. Some authors distinguish two components of resilience: resilience to destruction, that is, the ability to protect one’s own integrity under pressure, and, beyond resistance, the ability to forge a positive life behavior despite difficult circumstances [18]. It seems that resilience is not a characteristic that some people possess and others do not possess at all [16] but rather, it is an internal characteristic that can be reinforced or weakened by environmental factors [19,20].

The sources that contribute to the level of resilience are often grouped into two categories: external and internal [21]. The internal elements involve self-esteem, self-control, self-efficacy, and an internal locus of control [22], whereas the external elements producing resilience include, although they are not the only ones, social support and supportive environments, positive peer relationships, and a sense of belonging to a group [23]. The results of Montero-Carretero, and Cervelló [24] in a study with Spanish adolescents between the ages of 11 and 15, showed that the possibility of reporting and asking teachers for help, as well as the connection with the school, positively predicted resilience and this, in turn, negatively predicted victimization.

Regarding the relationship between resilience and bullying, few studies have analyzed the mediation effect of resilience on the victimization process. The work performed by Navarro, et al. [25] with adolescent students found that resilience had a moderating effect on victimization by cyberbullying, such that it was shown to be a “protector” against victimization, in line with the results of another study that analyzed the predictive power of resilience on cyberbullying [26] in 377 Turkish students aged 15 to 19, in which a simple regression analysis revealed that resilience negatively predicted cyberbullying perpetration and cybervictimization. The results of Hinduja and Patchin [16], in another work performed with American students aged 12 to 17, indicated resilience as a powerful protective factor, both to prevent experiences of intimidation and to mitigate their effect, in line with the claims of other authors [8] who already suggested the possible protective nature of resilience in such situations, or as in the work of Moore and Woodcock [27], in which students with poorer resilience were more prone to engage in bullying behaviors as well as to become victims. Another study in Israel [28] analyzed the impact of cultural settings and individual protective attributes on peer bullying and victimization in school. This study, involving 112 Jewish and 55 Bedouin Arab students aged 10 to 11, considered self-esteem, a sense of autonomy, emotion regulation, and individual resilience as protective attributes, and the authors concluded that resilience could play a more important role in predicting adolescent violence in school than other personality attributes. In the study of the relationships between resilience and bullying, the work of Tobias and Chapanar [29] proposed another perspective, analyzing how cyberbullying experiences can influence student resilience. The authors concluded that, whereas these cyberbullying events can be very traumatic for students, they sometimes produce increases in some resilience factors. This is easier as long as other social factors contribute to overcoming the events from appropriate climates.

On the other hand, the literature has described the importance of teacher instructional practices when generating positive school climates, characterized, among other things, by developing environments of respect and security with a high quality of social relations [30,31]. In this regard, some spaces within the school have special characteristics that could be more conducive to the development of bullying behaviors. Such is the case of the PE classroom, where the public demonstration of students’ motor skills is demanded, and where schoolchildren’s physical characteristics emerge in a particular way, and these are some of the main causes of bullying [32]. In this area, there is growing interest to study how the teacher’s interpersonal style will have a psychological impact on the students’ experiences [33]. For years, researchers concerned about pedagogy in PE have tried to discover the benefits of different teaching styles [34]. A review article that referred to the Spectrum of Teaching Styles [35] to elucidate the advantages of the different teaching styles concluded that “*all styles are valid pending the objectives of the experience, and each contributes to the overall educational process*” ([36], p. 202), although some works showed benefits in the social and moral domain of the styles that gave the student greater autonomy versus more directive styles [37,38,39]. The results of another review that focused on analyzing the current situation of PE-student-centered styles and methodologies at pre-university levels showed that teachers mostly use traditional or teacher-centered styles [40], although the Spanish education system indicates the need to implement student-centered styles for competence acquisition. The authors highlight the important contributions of psychology applied to teaching when justifying the change towards student-centered styles, especially from the self-determination theory (SDT).

The SDT [41,42] has been one of the main theoretical frameworks for explaining the behaviors of human beings based on their motivations, serving as a reference for analyzing teachers’ interpersonal styles. From this theory, and within the school context, teachers are considered very decisive social agents for the students to satisfy their three basic psychological needs (BPNs) of autonomy (acting freely without imposition), competence (feeling effective) and relatedness (connection with other people), which leads to more self-determined motivations that result in more adaptive consequences. Reeve [43] stated that, in PE classes, the most prominent source of support for students’ needs is the teacher’s motivating style. From this theoretical framework, the most studied teaching styles have been the autonomy support (AS) style and the controlling style (CS).

Referring to AS, Roth, et al. indicate that “*the contexts of support for autonomy involve the recognition of the child’s feelings, the adoption of the child’s perspective, justification, the possibility of choice, and the minimization of pressure*” ([44], p. 656). For their part, Guay and Vallerand defined the teacher’s AS as “*the degree to which people use techniques that encourage choice and participation in school activities*” ([45], p. 215). Following this approach, Tilga, et al. [46] propose that autonomy-supportive behavior could be characterized by three dimensions: organizational, procedural, and cognitive. Thus, organizational autonomy support encourages students to take possession of the environment and could include teaching behaviors that offer students opportunities to choose the teaching methods and where to perform an exercise. Procedural autonomy support encourages students to become the owners of the way activities are performed and could include teaching behaviors such as offering students the choice of how to present homework. Cognitive autonomy support encourages student learning and could include teaching behaviors such as asking the students to justify and defend their point of view, or to seek solutions to a problem.

In particular, and from the conceptual model of the self-determination theory [41,42], research on the PE teachers’ interpersonal styles supporting student autonomy has shown that such support has a positive effect on self-determined motivation in PE students, positively influencing affective and behavioral relationships in class [46,47]. Other studies have shown positive relationships between AS and prosocial behavior [48], and a greater intention to be physically active [49]. In a study conducted with students in PE classes, Lam, et al. [50] showed that AS negatively predicted bullying behaviors through satisfaction of the need for relatedness. Roth, et al. [44] observed an inverse relationship between the AS perceived by students and bullying behavior, and they suggest that schools should establish AS teacher styles, and that researchers should continue to analyze the effect of this interpersonal teacher style on bullying behaviors., a recent study in South Korea [51] analyzed the effects of a training program, called Autonomy-Supportive Intervention Program (ASIP), aimed at increasing the autonomy-support of PE teachers from the SDT framework. Among the different contents of the program, PE teachers learned to implement six recommended AS instructional behaviors, including taking the students’ perspective, vitalizing inner motivational resources, using informational language, providing explanatory rationales, acknowledging and accepting negative affect, and displaying patience. ASIP participation increased teachers’ AS and students’ need satisfaction and prosocial behavior, and it decreased teachers’ control and students’ need thwarting, antisocial behavior, and positive attitude toward cheating. Systematic review of the effects of AS-based programs in PE [52] showed positive effects of AS on the satisfaction of students’ BPNs and self-determined motivations, and concluded by emphasizing the importance of PE teachers developing this interpersonal style to encourage physical exercise outside the school and to avoid a number of negative and maladaptive behaviors of students in the PE classroom.

On the other hand, CS is defined by authoritarian attitudes of the teacher, who ignores the students’ preferences and tries to impose a specific way of thinking, feeling, and behaving, using pressure [43,53]. Teachers who teach with this style sometimes resort to shouting, threatening, or punishing to gain control of the students, and at other times, they try to provoke feelings of guilt or embarrassment in the student, withdrawing their attention or interest and expressing disappointment when their expectations are not met [54].

In contrast to the AS, teachers’ CS has shown relationships with lower levels of perceived satisfaction and student commitment [55], an increase in levels of physiological stress markers, such as cortisol [56], and increases in students’ anger and anxiety [57]. For instance, teacher controlling style, primarily through the intimidation factor, significantly predicted bullying and anger in a sample of 602 adolescent students [58].

Both resilience and the teacher’s interpersonal style seem to be related to the emergence and regulation of bullying behaviors. However, the interaction between these factors and the extent to which the PE teacher’s interpersonal style can predict such behaviors remain to be studied. Therefore, the analysis of the aforementioned interaction was the main objective of our work.

Some studies have highlighted the need to develop measuring instruments that can collect, in the field of PE, both the AS and the CS style of the PE teacher. Thus, in the work of Tilga, et al. [59], the need to measure the different components of autonomy support has been highlighted, distinguishing between cognitive, organizational, or procedural autonomy. That is why validation of these instruments in new languages and cultures is necessary. There is also a need to develop or adapt instruments for the measurement of resilience that are suitable for PE. For the measurement of resilience in Spanish, the existing measuring instruments [25] had never been subjected to a confirmatory factorial analysis, so it is necessary to validate them. That is why, as a secondary objective, the Spanish versions of the measuring instruments of the studied variables will be validated, so that they can be useful for the evaluation of the teacher’s interpersonal style and of student resilience.

Our working hypotheses are:

(1) PE teacher’s interpersonal AS style will be positively related to and will positively predict resilience, considering the characteristics of an interpersonal style based on AS [45,46] and external sources that facilitate the development of resilience, such as social support and supportive environments, positive peer relationships, and a sense of belonging to a group [21,23]. In turn, PE teacher’s interpersonal AS style will negatively predict bullying behaviors, as was the case in previous studies [44,49,50] in which this interpersonal style negatively predicted antisocial behavior [51].
(2) The CS will be positively related to and will positively predict bullying behaviors as in a study with Estonian students between 12 and 16 years [58].
(3) Resilience will be negatively related to and will negatively predict victimization as in previous studies on traditional bullying [24,25,27,28] and cyberbullying [26].

## 2. Materials and Methods

### 2.1. Sample

The necessary sample size for our study was calculated. The confidence level of 95%, an error of 5% and a probability of 50% were established to calculate the sample size. With these characteristics, the minimum sample size required was 385 cases. Five-hundred and thirty-seven students from public and concerted (seven public and five concerted) schools of the province of Alicante (Spain) participated in the study, of whom 69 students were in 6th grade of Primary School (32 boys and 37 girls), 319 were students of 1st grade of Compulsory Secondary Education (CSE) (167 boys and 152 girls), and 149 students were in 2nd grade of CSE (62 boys and 87 girls). Students were aged between 11 and 15 (M = 12.91, SD = 1.91). The χ^2^ test that was carried out to check the distribution by age and gender was nonsignificant (χ^2^ (4) = 6.598; *p* < 0.160). The sample was a convenience sample. The participating schools were chosen by the competent educational authority, authorizing specific schools. In fact, the authorization file indicated this. We measured in the age groups of our interest in all those schools for which we received authorization.

### 2.2. Measuring Instruments

The following measuring instruments used to analyze the study variables:

#### 2.2.1. Measurement of the Physical Education Teacher’s Autonomy Support and Controlling Style

We used an adaptation of the Multidimensional Perceived Autonomy Support Scale for Physical Education (MD-PASS-PE) by Tilga, et al. [46] and of the Controlling Style factor of the Empowerment in Sport Questionnaire (EDMCQ-C) by Appleton, et al. [60]. This scale consists of 19 items grouped into 4 factors (Organizational Autonomy Support (five items), Procedural Autonomy Support (five items), Cognitive Autonomy Support (five items), and Teacher Controlling Style (four items). The answers are rated on a seven-point Likert scale ranging from 1 (totally disagree) to 7 (totally agree). The items refer to various psychosocial competences linked to the teacher and the teacher-created climate in PE classes.

From this questionnaire, a General Autonomy Support Factor is obtained by calculating the mean of the three autonomy support factors. The original authors of the questionnaire recommend using this approach when performing mathematical prediction processes, to avoid problems of co-linearity and variance inflation [46].

The items related to Organizational Autonomy Support describe situations such as: “My PE teacher allows me to exercise in different ways.” An example item related to Procedural Autonomy Support is “My PE teacher helps students find solutions.” Items related to Cognitive Autonomy Support refer to situations such as “My PE teacher takes into account what the students want to do.” Finally, the items related to the Controlling Style describe situations such as: “My teacher is less friendly toward students who do not see things his/her way.”

#### 2.2.2. Resilience Measurement

The Spanish version of the 10-item Resilience Scale (CD-RISC) by Connor and Davidson [61] was used to measure this variable. The scale refers to various personal psychological skills. It is rated on a five-point Likert items, ranging from 1 (never) to 5 (almost always). Each item is formulated through direct statements in the first person. The items describe situations such as: “*I know how to adapt to changes, I see the positive side of things...*” This scale has been adapted to the educational environment [16]. Although there exists a Spanish version of the scale [62] for adults, which has been used for adolescents [25], we do not know of any studies that have tested its psychometric properties in Spanish, except for its reliability [25].

#### 2.2.3. Measurement of Bullying

The Spanish version the European Bullying Intervention Project Questionnaire (EBIP-Q) validated by Ortega-Ruiz, et al. [63] was used. This scale includes two factors, which reflect bullying behaviors (victimization and bullying), with seven items each. The first seven items are related to victimization and describe situations such as: “*Someone has robbed me or broke my things, someone has threatened me…*” The last seven items are related to bullying and describe situations such as: “*I have stolen or damaged someone’s property, I have threatened someone…*” Students are asked to indicate how often they have performed or suffered these behaviors in the past two months. Each item is formulated through direct questions in the first person. The student must answer them on a five-point Likert scale, as follows: 1 (No), 2 (Yes, once or twice), 3 (Yes, once or twice a month), 4 (Yes, about once a week) to 5 (Yes, more than once a week). In a validation study [63], confirmatory factor analysis (CFA) found very strong fit indices, showing the robustness of the instrument.

### 2.3. Procedure

First, a random cluster sample was selected of the schools of the province of Alicante. The school directors were then contacted to encourage them to participate and inform them about the objectives of the study, as well as its exclusively scientific and academic purposes. In addition, they were informed of the voluntary nature of the test, and the strict confidentiality of the data obtained therein. Once the school directors had agreed, a written statement was sent to request the informed consent of the parents and of the Autonomous Secretariat of Education, which gave its authorization (file 05ED01Z/2017. 56).

After obtaining the necessary permits and authorizations, the teachers in charge were coordinated on the day of the surveys. PE teachers were previously instructed by the researchers to administer the questionnaires appropriately. Data collection was carried out in a classroom of each school in one of the hours scheduled for PE class during the first trimester. Prior to the test, students were instructed about the importance of responding sincerely. During the completion of the questionnaires, any doubts that arose were clarified by the teacher of the subject. The questionnaires were completed in approximately 20 min.

### 2.4. Data Analysis

#### 2.4.1. Validation and Reliability of the Measuring Instruments

For the validation of the measuring instruments that had not been previously validated in the Spanish educational context, (The Physical Education Teacher’s Autonomy Support and Controlling Style Scale and the Resilience Scale) CFA was used. In those instruments that had already been validated (European Bullying Intervention Project Questionnaire) reliability was calculated.

The maximum likelihood estimation method was used for CFA and, following the recommendations of Hu and Bentler [64], a combination of several fit indices was used to contrast the adequacy of the proposed models. Specifically, the ratio between chi squared and degrees of freedom (χ^2^/df), the Comparative Fit Index (CFI), Incremental Fit Index (IFI), Root Mean Square Error of Approximation (RMSEA) plus its 90% confidence interval (CI), and the Standardized Root Mean Square Residual (SRMR) were used. For the χ^2^/df coefficient, values below 3 are generally considered acceptable, although some more conservative authors accept values below 5 [64]. RMSEA values equal to or less than 0.08 and SRMR values equal to or less than 0.06 are considered acceptable. However, there is a widespread consensus to consider that these values are only indicative [65], and that for indices such as the CFI and IFI, more conservative criteria (values equal to or greater than 0.90) are considered an acceptable lower limit. In short, as Cea [66] indicates, rather than a single value, a global exploration of all values must be performed to accept the validity of a measuring instrument.

Factorial invariance was tested using the procedure outlined in Byrne [67]. First, the baseline model was tested (Step 1). This model’s fit to the data was acceptable. The second step was the verification of the metric invariance (pattern coefficients) and the measurement invariance (latent factors) across groups [67]. First we calculate a model unconstrained for gender and age respectively. Then, we calculate a model in which only the factor loadings (i.e., measurement weights) are constrained equal across group, a model in which all estimated factor loadings, as well as factor variances and covariances (i.e., structural covariances), are constrained equal across groups, and a final model having all estimated factor loadings, factor variances, factor covariances, and error variances (i.e., measurement residuals) constrained equal across groups. The difference in CFI between the unconstrained model and the models in the invariance routine was considered as a criterion to accept invariance. Values less than 0.01 are considered acceptable according to the criterion established by Cheung and Rensvold [68] to support model invariance. In addition, the model was compared without constraints with the other models of invariance through the significance of χ^2^ When the difference between the unconstrained model and the constrained models is nonsignificant, the model is considered invariant [69,70]. With regard to reliability analyses, the Cronbach alpha coefficient was used. This coefficient can be considered acceptable when it is greater than 0.70 [71].

#### 2.4.2. Relationship of Bullying Behaviors with Resilience and Teacher’s Autonomy-Supportive or Controlling Style

**Figure 1:**
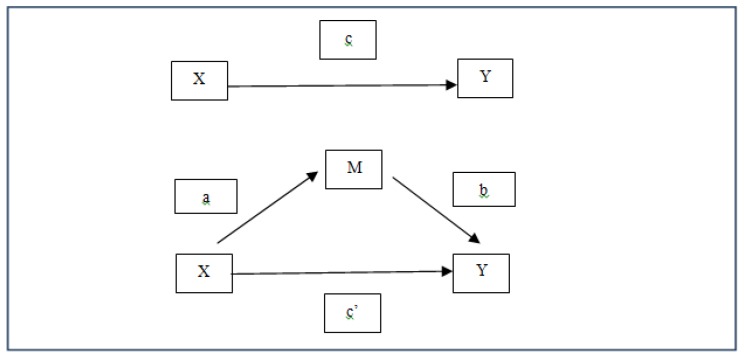
Graphic representation of a simple mediation model [72].

To analyze the relationship between bullying and personal (resilience) and social factors (PE teacher’s autonomy supportive and controlling style), in addition to the study of descriptive statistics, Pearson’s simple correlation, multiple regression analysis, and simple mediation models were calculated to explore the indirect effect of some variables on bullying behavior (see Figure 1). Mediation models are especially useful when, despite the fact that regression analyses do not show significant prediction, one wishes to explore whether the effect of a predictor variable (X) on a predicted variable (Y) can be mediated by a third variable (M).

The mediator or indirect effect can be calculated both by the difference between the total and the direct effect (c-c’), and by the product of the coefficients a and b (a × b). There are different ways to calculate the statistical significance of this effect. We used the latest bootstrapping method, with techniques for simple mediation. We used the “Process” macro of Preacher and Hayes [73], with 10,000 samples, calculating the 95% CI of the indirect effects. According to this methodology, mediation exists when the CI does not include zero. The SPSS 25 programs, with the macro Process, and Amos 18 were used to perform all statistical calculations.

## 3. Results

### 3.1. Confirmatory Factor Analysis (CFA)

#### 3.1.1. Physical Education Teacher’s Autonomy-Supportive and Controlling Style Scale

A model (Table 1) was tested with three first-order factors (Organizational Autonomy Support, Cognitive Autonomy Support, and Procedural Autonomy Support) and one second-order factor (General Autonomy Support), along with the Controlling Style Factor. The different fit indices analyzed showed poor fit values for the proposed model (Model 1). Inspection of the factor loadings revealed that Item 9 presented fit problems, so this item was removed, and CFA was recalculated (Model 2), finding that the model now fit correctly. Thus, Model 2 was considered the final acceptable Model. For the PE Teacher’s Autonomy-Supportive and Controlling Style Scale, the comparison of models between the unconstrained model and the others constrained models with the CFI showed that the models were invariant by gender and age (ΔCFI < 0.01). In addition, the chi squared statistic showed that, when comparing the unrestricted model with the measurement weights model, the structural weights model, and the structural residuals model the difference was nonsignificant both for age (χ^2^ (14) = 19.793, *p* < 0.138 for the measurement model, χ^2^ (16) = 24.528, *p* < 0.08 for the structural weights model and χ^2^ (22) = 34.158, *p* < 0.052) for the structural residuals model), and gender invariance. In the case of age, the comparison values were χ^2^ (28) = 35.226, *p* < 0.164 for the measurement model, χ^2^ (32) = 39.478, *p* < 0.170 for structural weights model and χ^2^ (44) = 58.919, *p* < 0.066) for the structural residuals model). With regard to the analysis across gender and age, no significant differences were found in the model χ^2^ between the unconstrained model and the model in which the factor loadings were set as invariant, which is a minimum criterion for invariance [69].

#### 3.1.2. Resilience Scale

The analysis of the Resilience Scale revealed the existence of a single factor and showed that the model fit correctly, as all the fit indices amply exceeded the recommendations. For the Resilience questionnaire (Table 2), the results also showed increments lower than 0.01 for the comparison of CFI between the invariance models. In addition, the comparison of the chi square statistic showed nonsignificant values both for gender invariance (χ^2^ (9) = 10.316, *p* < 0.326 for the measurement model, and χ^2^ (10) = 10.448, *p* < 0.403 for the structural weights model), and for age invariance (χ^2^ (18) = 13.724, *p* < 0.748 for the measurement model and χ^2^ (20) = 16.392, *p* < 0.692 for the structural weights model). The questionnaire was also shown to be invariant by gender and age, corroborating its robustness (Table 2).

### 3.2. Descriptive Statistics, Reliability Analysis, and Correlation Analysis

Table 3 shows the optimal reliability values based on the internal consistency coefficients of the factors (Cronbach alpha), which were above 0.79 in all cases [71]. According to the results of the correlation analyses, a moderate correlation between bullying and victimization was observed. To a lesser extent, we also found a positive correlation between Resilience and General Autonomy Support. We also obtained significant correlations between the Controlling Style and the two bullying behaviors (Victimization and Bullying). It should be noted that very consistent relationships were found between Bullying and Victimization. Finally, the bullying factor was the only one that had a negative correlation with General Autonomy Support (see Table 3).

In addition, due to the continuous nature of the Bullying and Victimization variables and to verify whether there were differences in the correlations when compared with Pearson’s correlations, Spearman’s correlation of these variables with the other factors was calculated. The results showed very similar results, to those found when calculating Pearson correlations. Thus, Victimization correlated positively with Bullying (Р = 504; *p* < 0.01), and with Controlling Style (Р = 0.168; *p* < 0.05). On the other hand, Bullying correlated positively with Controlling Style (Р = 0.242; *p* < 0.01) and negative with the Resilience (Р = −0. 211; *p* < 0.01), the Cognitive Autonomy Support (Р = −0.153; *p* < 0.01), Procedural Autonomy Support (Р = −0.164; *p* < 0.01), Organizational Autonomy Support (Р = −0.089; *p* < 0.05), and general the Autonomy Support (Р = −0.149; *p* < 0.01).

### 3.3. Prediction of Bullying (Bullying and Victimization) Based on Personal and Situational Factors

#### 3.3.1. Regression Models

The results in Table 4 show that bullying was predicted very significantly and positively by the PE teacher’s Controlling Style and negatively by the teacher’s General Autonomy Support. In the model predicting bullying from Resilience, General Autonomy Support, and PE Teacher’s Controlling Style, these variables explained 7.4% of the variance of bullying.

Regarding the model of prediction of Victimization, we found significant regression weights for all the variables (with 5% explained variance of victimization), and in the expected direction, except for General Autonomy Support, which, contrary to our hypotheses, did not predict Victimization negatively (see Table 5).

#### 3.3.2. Mediation Model: General Autonomy Support (X)–Resilience (M)–Victimization (Y)

**Figure 2:**
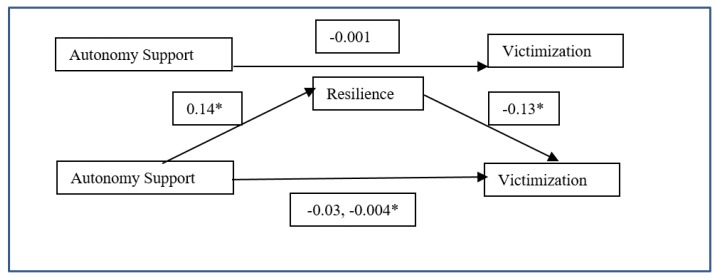
Mediation model of autonomy support on victimization through resilience (* *p* < 0.05).

As the value of the regression analysis of Autonomy Support on victimization was not statistically significant, a mediation model was calculated to explore whether victimization could be indirectly predicted by Autonomy Support, with resilience mediating this relationship. The results of this model indicated a 95% CI with values of −0.03 and −0.004. As the null value (0) was not found within the CI, it was concluded that the indirect effect was significant. In addition, as the regression analysis had indicated, General Autonomy Support predicted resilience positively, and resilience predicted victimization negatively (Table 6 and Figure 2).

## 4. Discussion

The objective of this study was to analyze the relationships between the PE teacher’s interpersonal style, students’ resilience, bullying, and victimization. Whereas some studies have shown the relationship between PE teachers’ interpersonal style with bullying and victimization, and some others have described negative relationships between resilience and bullying and victimization, we know of no studies to date that have analyzed the role of resilience in relation to the interpersonal style of the teacher of any educational subject and bullying. Therefore, the analysis of all the variables under study in our work makes it a pilot study, which could be considered a relevant contribution. In addition, the study of these variables in the specific context of PE may be of particular interest when considering the responsibility that the Spanish education system gives to this subject on values education and the development of social and citizen competence [11].

In favor of our first hypothesis, AS in PE classes positively predicted students’ resilience. These results give the PE teacher the opportunity to contribute to the promotion of more resilient students, generating supportive climates, fostering positive peer relationships, and increasing the feeling of belonging to the group, which, as suggested by other authors, is critical for the development of resilience [21,23]. Also in favor of the first hypothesis, the AS provided by the PE teacher reduced bullying, as previously occurred in [50] and just as it did with 13- and 14-year-old Israelis, where the AS provided by teachers from other knowledge areas was decisive in the internalization of pro-social values, to the detriment of bullying behavior [44]. According to these authors, programs that reduce the frequency of bullying predominantly through rigorous control of the setting are only effective while such control remains, but cease to be so when control is not so clearly manifest. On the other hand, the advantage of programs which, through AS, promote the identification and integration of pro-social values and behaviors is that the effects of the program are maintained over time and everywhere. This seems to be because young people decrease their aggressions due to their own appraisal of such behaviors, and not because they feel they are in a setting where such behaviors are will be punished. Readers are reminded that one of the challenges that program creators face when they design programs to prevent bullying is to make their effects last over time [10]. In addition, the results of this study showed that CS favored more student bullying, confirming the second hypothesis, and these results are similar to the findings of Hein, et al. [58].

On the other hand, in relation with the predictive nature of PE teachers’ interpersonal styles on the complex phenomenon of bullying, we expected that both AS and CS would predict victimization, as occurred with bullying. However, only the CS, together with resilience (the former positively and the latter negatively) predicted victimization. Contrary to our expectations, AS did not do so directly. It has already been shown that CS within the educational realm have very negative maladaptive consequences, such as anger, anxiety, perceived low satisfaction, or lack of commitment [55,56,57]. A review [52], whose objective was to analyze the effects of interpersonal styles in PE, showed that CS was associated with high levels of amotivation [74,75,76,77,78]. Since, contrary to our initial hypotheses, AS did not appear to be a significant predictor of victimization, we decided to study in more depth the relationships of these constructs and, through mediation analysis, seek possible underlying relationships. In this analysis, AS predicted resilience and this predicted victimization, in favor of the third hypothesis, in which this predictive power of resilience on victimization was expected. This approach, through mediation analysis, was decisive in understanding the influence of AS on victimization, mediated by resilience. In view of these results, it would be interesting to continue to study the role of resilience in the face of victimization behaviors, and, as mentioned, to make young people resistant to stressful situations and to help them generate adaptive responses that will make them stronger after these events. Zych, et al. [10] state that future work should shed light on mechanisms that build resilience, and suggest that intervention programs aimed at preventing bullying should incorporate strategies to increase social and emotional competence, and recommend changing the educational curriculum to address these contents. In the same direction Beltrán-Catalán, et al. [79] recommend further research on how emotional intelligence can help in the face of victimization. In fact, the results of Casas, et al. [80] identified as predictors of victimization factors such as the low ability to distinguish one’s emotions at any given time and some people’s low perception of their ability to replace their negative emotions with more positive ones. Considering these recommendations, the relationships of resilience with school engagement [81], and our results, we propose that formulas be included in the subject of PE to make students more resilient. We note that the scenario presented by this subject could be an ideal environment to detect cases of bullying early on so as to intervene in prevention [32]. Some authors have already shown that sport and physical activity can be an ideal means to build personal resilience through the improvement of physical self-concept [82], so damaged in victims of bullying [83].

In this direction, and considering the findings of Del Rey, et al. [84] that point to the strong effect that teaching management has on emotions and the emergence of bullying or victimization behaviors, it seems increasingly advisable for teachers to train and apply styles of AS [85], avoiding CS [86]. Given the works that indicate the need for more evidence to demonstrate the benefits of student-centered teaching styles over more traditional and directive ones, the results of this study seem to support those of other authors who have previously shown better results in the social and moral levels when using these styles that facilitate students’ choice [37,38,39]. Thus, considering the results of Zapatero [40], who stated that PE teachers currently exercise predominantly traditional teacher-centered methodologies, we emphasize the importance of PE teachers’ being trained in styles that promote autonomy. These specific trainings have provided good results in previous programs [51], achieving increased teachers’ AS and students’ need satisfaction and prosocial behavior, and they decreased teachers’ control and students’ need thwarting and antisocial behavior.

Thus, from a practical point of view, and in view of our results, it would be very interesting if, among other things, PE teachers could ask students for feedback on their tastes and preferences concerning the exercises or games that serve to achieve specific objectives, taking this into account when planning classes. They could also give students a choice of the classmates with whom to form groups to perform tasks, how to perform some of the tasks, the time they want to spend on each one, the order in which they perform them, the material with which to perform them, or the location within the available school spaces where they do them. It may even be advisable to assign responsibility to the students in the evaluation process, as suggested by Moreno-Murcia, et al. [87]. The fact that students perceive that the teacher gives them the opportunity to choose within their classes does not clash with the achievement of the goals set by the teacher, provided that the teacher has sufficient training in the subject taught as well as in teaching methodologies that allow the development of an interpersonal style far from the more traditional, controlling, and dictatorial styles within the classroom [88]. In addition, motor games within the PE class offer a multitude of possibilities to manage conflict in a safe and regulated environment. With the teacher’s help, students can establish a relationship between the challenges and conflicts of games during the classes and those they encounter in everyday life in relation to bullying, contributing to the development of resilience in students for whom these challenges imply some stress and self-improvement. On the other hand, cooperative games, in which the teacher proposes the achievement of joint objectives to groups of students, could be ideal for improving relations among them, taking into account the benefits of cooperative learning [89].

In this sense, and from the subject of PE, the opposing positions among students participating in direct opposition games, where the achievement of the objective by some of them implies dismantling the opponents’ strategies, imposing their own potentials (fighting for a space, tug of war, controlling the opponent’s body according to sports rules such as judo, trying to score in two-on-one situations, etc.), can be the ideal means for students to train in the control of their emotions by confronting the controlled encounters that may arise from such situations within the classroom [90].

Thus, we consider that the PE teacher, showing his/her responsibility as a teacher and through styles with AS, has the opportunity to contribute to the development of the students’ personal resilience and to the prevention of bullying behaviors through a multitude of proposals, ranging from exercises and games where young people collaborate to overcome challenges, to those that require maximum opposition between them.

Regarding the second objective of the study, the statistical analyses carried out have shown that the Spanish versions of the Multidimensional Perceived Autonomy Support Scale for Physical Education (MD-PASS-PE) by Tilga, et al. [46] and of the Controlling Style factor of the Empowerment in Sport Questionnaire (EDMCQ-C) by Appleton, et al. [60], and the Spanish version of the 10-item Resilience Scale (CD-RISC) by Connor and Davidson [61] are robust to measure the intended constructs and are consistent in their relationship with the other constructs analyzed, so they can be used in PE. As in the work of Tilga, et al. [59], the questionnaire is invariant (in this case, by age and group), obtaining fit indices very similar to ours.

## 5. Conclusions

The interpersonal styles of AS and CS of PE teachers predicted resilience as well as bullying and victimization. In addition, resilience directly predicted bullying, acting as a mediator between autonomy support and victimization. This study provides useful information for teaching professionals, highlighting the benefits of interpersonal styles based on PE teachers’ AS versus CS to decrease bullying behaviors and increase student resilience. The results increase the body of knowledge about external sources that contribute to the development of resilience, and show the role that resilience can play as a direct predictor of bullying behaviors, meeting the demands other researchers suggesting the need for further research in this particular regard [10]. Although the results should be taken with caution, considering that the explained variance of bullying and victimization in the different models is small, the mediating nature of resilience in the relationships between the AS style and victimization is a relevant contribution, as no previous studies are known that have demonstrated this before. Similarly, future studies should include teachers’ perceptions of these behaviors in students as well as the effect that training programs in teaching styles might have on the decrease of bullying behaviors.

### Limitations and Future Prospects

However, we can consider as a limitation the exclusive use of correlational methodology, which does not allow establishing causal relationships or examining the described processes in depth, as would experimental and qualitative methodologies. It is therefore proposed that future studies address this aspect from the above-mentioned methodological approaches and, in addition, corroborate in other samples the robustness of the measuring instruments used herein. Also, with regard to the second objective, it would be necessary to develop specific measuring instruments for the field of PE (in the case of the teacher’s CS factor or resilience), rather than using adaptations of other instruments developed in areas other than PE.

On the other hand, in view of our results, we suggest the inclusion of statistical analyses of mediation and moderation, which would help to reveal still concealed relationships between the variables related to the subject under study. Considering that, in this study, the variables were measured at a single moment, which is a methodological limitation, we propose that future studies replicate with other samples the analysis of the model presented using longitudinal designs, measuring at two or three different moments.

## Figures and Tables

**Table 1:** Confirmatory factor analysis and multi-group invariance analysis of the Physical Education Teacher’s Autonomy-Supportive and Controlling Style Scale.

Models	χ^2^	*df*	χ^2^/*df*	CFI	IFI	SRMR	RMSEA	90% CI
Model 1	812.282	148	5.448	0.897	0.897	0.073	0.092	0.085–0.098
Model 2	655.556	131	5.004	0.915	0.915	0.066	0.086	0.080–0.093
Model 3	839.797	262	3.205	0.906	0.907	0.073	0.064	0.059–0.069
Model 4	859.590	276	3.114	0.905	0.906	0.079	0.063	0.058–0.068
Model 5	864.325	278	3.109	0.905	0.905	0.080	0.063	0.058–0.068
Model 6	872.621	281	3.105	0.905	0.904	0.080	0.063	0.058–0.068
Model 7	873.955	284	3.077	0.905	0.904	0.080	0.062	0.058–0.068
Model 8	923.163	302	3.057	0.899	0.899	0.080	0.062	0.058–0.067
Model 9	992.550	393	2.526	0.904	0.905	0.066	0.053	0.049–0.058
Model 10	1027.776	421	2.441	0.904	0.904	0.066	0.052	0.048–0.056
Model 11	1032.027	425	2.428	0.904	0.903	0.066	0.052	0.048–0.056
Model 12	1046.801	431	2.429	0.903	0.903	0.070	0.052	0.048–0.056
Model 13	1051.468	437	2.406	0.903	0.903	0.071	0.051	0.047–0.055
Model 14	1189.941	473	2.516	0.885	0.885	0.073	0.053	0.050–0.057

*Notes*: Model 1 = Baseline model with three first order factor and a second order factor for Autonomy Support and a single factor for Controlling Style with 19 items; Model 2 = Baseline model with three first order factor and a second order factor for Autonomy Support and a single factor for Controlling Style with 18 items (minus item 9); Model 3 = invariant gender model unconstrained; Model 4 = invariant gender model measurement weights; Model 5 = invariant gender model structural weights; Model 6 = invariant gender model structural covariances; Model 7 = invariance gender model structural residuals; Model 8 = invariant gender model measurement residuals; Model 9 = invariant age model unconstrained; Model 10 = invariant age model measurement weights; Model 11 = invariant age model structural weights; Model 12 = invariant age model structural covariances; Model 13 = invariant age model structural residuals; Model 14 = invariant age model measurement residuals.

**Table 2:** Confirmatory factor analysis and multi-group invariance analysis of the Resilience Scale.

Models	χ^2^	*df*	χ^2^/*df*	CFI	IFI	SRMR	RMSEA	90% CI
Model 1	74.110	34	2.180	0.961	0.962	0.034	0.047	0.032–0.062
Model 2	141.477	68	2.081	0.932	0.933	0.052	0.045	0.034–0.055
Model 3	151.79	77	1.971	0.932	0.931	0.051	0.043	0.033–0.053
Model 4	151.925	78	1.948	0.931	0.932	0.053	0.042	0.032–0.052
Model 5	162.634	102	1.594	0.943	0.946	0.048	0.033	0.023–0.043
Model 6	176.358	120	1.470	0.943	0.946	0.049	0.030	0.020–0.039
Model 7	179.026	122	1.467	0.947	0.947	0.050	0.030	0.022–0.039

*Notes*: Model 1 = Baseline model with one-factor model with 10 items; Model 2 = Invariant gender model without restrictions; Model 3 = Invariant gender model factorial weights; Model 4 = Invariant gender model structural covariances; Model 5 = Invariant age model without restrictions; Model 6 = Invariant age model factorial weights; Model 7 = Invariant age model structural covariances.

**Table 3:** Descriptive statistics, reliability analyses and correlations with bullying behaviors, resilience and autonomy support and controlling style in physical education.

Variables	MD	SD	α	1	2	3	4	5	6	7	8
1. Victimization	1.47	0.59	0.79	1	0.522 *	−0.157 **	−0.040	−0.031	−0.031	0.170 **	−0.037
2. Bullying	1.23	0.42	0.80		1	−0.079	−0.132 **	−0.120 **	−0.051	0.247 **	−0.109 *
3. Resilience	3.36	0.69	0.79			1	0.254 **	0.269 **	0.231 **	−0.083	0.272 **
4. Cognitive Autonomy Support	5.10	1.47	0.90				1	0.867 **	0.750 **	−0.019	0.948 **
5. Procedural Autonomy Support	5.47	1.36	0.88					1	0.696 **	−0.090	0.924 **
6. Organizational Autonomy Support	4.44	1.47	0.82						1	0.111	0.889 **
7. Controlling Style	2.61	1.49	0.82							1	0.003
8. General Autonomy Support	5.00	1.32	0.89								1

* *p* < 0.05. ** *p* < 0.001.

**Table 4:** Coefficients of the model predicting bullying from resilience, physical education teacher’s overall autonomy support and controlling style.

Coefficients					
Model	Non-Standardized Coefficients		Standardized Coefficients	*t*	*p*
	B	Standard Error	β		
(Constant)	1.285	0.104		12.313	0.000
Resilience	−0.019	0.026	−0.031	−0.721	0.471
Controlling Style	0.069	0.012	0.244	5.841	0.000
General Autonomy Support	−0.032	0.014	−0.101	−2.340	0.020

**Table 5:** Coefficients of the model predicting victimization from resilience, the physical education teacher’s general autonomy support and controlling style.

Coefficients					
Model	Non-Standardized Coefficients		Standardized Coefficients	*t*	*p*
	B	Standard Error	β		
(Constant)	1.723	0.149		11.588	0.000
Resilience	−0.123	0.038	−0.144	−3.269	0.001
Controlling Style	0.062	0.017	0.158	3.720	0.000
General Autonomy Support	−0.001	0.020	−0.001	0.033	0.974

**Table 6:** Results of the total, direct, and indirect effects of the mediation model between Overall Autonomy Support, and victimization, mediated by resilience.

Predictor Variable	Criterion Variable	Total Effect		Direct Effect		Indirect Effect Path c’				Path a		Path b	
		Est.	SE	Est.	SE	Est.	SE	95% CI		Est.	SE	Est.	SE
								Lower	Upper				
Autonomy Support	Victimi-zation	−0.01	0.19	−0.00	0.01	−0.01	0.00	−0.03	−0.004	0.14 *	0.02	−0.13 *	0.03

* *p* < 0.05
